# Pathogenicity and immunogenicity of attenuated, *nef*-deleted HIV-1 strains *in vivo*

**DOI:** 10.1186/1742-4690-4-66

**Published:** 2007-09-23

**Authors:** Paul R Gorry, Dale A McPhee, Erin Verity, Wayne B Dyer, Steven L Wesselingh, Jennifer Learmont, John S Sullivan, Michael Roche, John J Zaunders, Dana Gabuzda, Suzanne M Crowe, John Mills, Sharon R Lewin, Bruce J Brew, Anthony L Cunningham, Melissa J Churchill

**Affiliations:** 1 Macfarlane Burnet Institute for Medical Research and Public Health, Melbourne, Victoria, Australia; 2 Department of Microbiology and Immunology, University of Melbourne, Melbourne, Victoria, Australia; 3 Department of Medicine, Monash University, Melbourne, Victoria, Australia; 4 Department of Microbiology, Monash University, Melbourne, Victoria, Australia; 5 Department of Epidemiology & Community Medicine, Monash University, Melbourne, Victoria, Australia; 6 National Serology Reference Laboratory, St. Vincent's Institute for Medical Research, Fitzroy, Victoria, Australia; 7 Australian Red Cross Blood Service, Sydney, New South Wales, Australia; 8 Faculty of Medicine, University of Sydney, Sydney, New South Wales, Australia; 9 Center for Immunology, St. Vincent's Hospital, Sydney, New South Wales, Australia; 10 Department of Neurology, St. Vincent's Hospital, Sydney, New South Wales, Australia; 11 Dana-Farber Cancer Institute, Boston, MA, USA; 12 Department of Neurology, Harvard Medical School, Boston, MA, USA; 13 Infectious Diseases Unit, Alfred Hospital, Melbourne, Victoria, Australia; 14 Westmead Millennium Institute, Westmead, New South Wales, Australia

## Abstract

In efforts to develop an effective vaccine, sterilizing immunity to primate lentiviruses has only been achieved by the use of live attenuated viruses carrying major deletions in *nef *and other accessory genes. Although live attenuated HIV vaccines are unlikely to be developed due to a myriad of safety concerns, opportunities exist to better understand the correlates of immune protection against HIV infection by studying rare cohorts of long-term survivors infected with attenuated, *nef*-deleted HIV strains such as the Sydney blood bank cohort (SBBC). Here, we review studies of viral evolution, pathogenicity, and immune responses to HIV infection in SBBC members. The studies show that potent, broadly neutralizing anti-HIV antibodies and robust CD8+ T-cell responses to HIV infection were not necessary for long-term control of HIV infection in a subset of SBBC members, and were not sufficient to prevent HIV sequence evolution, augmentation of pathogenicity and eventual progression of HIV infection in another subset. However, a persistent T-helper proliferative response to HIV p24 antigen was associated with long-term control of infection. Together, these results underscore the importance of the host in the eventual outcome of infection. Thus, whilst generating an effective antibody and CD8+ T-cell response are an essential component of vaccines aimed at preventing primary HIV infection, T-helper responses may be important in the generation of an effective therapeutic vaccine aimed at blunting chronic HIV infection.

## Introduction

Despite considerable effort, all attempts to develop an effective human immunodeficiency virus (HIV) vaccine based on subunit or prime-boost strategies have failed to elicit sterilizing immunity and protect against infection with wild type virus (reviewed in [1-3]). Current World Health Organization estimates indicate 42 million people are infected with HIV and approximately 20 million have died from AIDS. Approximately 5 million new infections occur annually. The overwhelming majority of these individuals live in developing countries with little or no access to potentially lifesaving antiretroviral therapies. Moreover, HIV is predicted to become the leading burden of disease in middle and low income countries by 2015 [4]. Thus, the need for an effective preventative and/or therapeutic HIV vaccine has never been more urgent.

Since the discovery of HIV nearly 25 years ago, there have been significant advances in our knowledge of HIV immunology (reviewed in [5-7]). As early as 1990 subunit vaccines based on the HIV envelope protein were developed, based on the observation that vaccinated chimpanzees were protected against homologous HIV challenge [8]. However, it is unlikely that such vaccines will ever be able to illicit immune responses sufficient for protection against heterologous HIV strains and, in fact, these approaches have failed repeatedly in animal models. In addition, HIV envelope protein-based vaccines were not efficacious in 2 phase III vaccine trials in humans [9-12]. More sophisticated vaccine approaches have targeted cellular immunity by the development of DNA prime-boost strategies, and have achieved strong stimulation of antibody and cytotoxic T-lymphocyte (CTL) responses in monkeys. However, despite robust immunological responses, these strategies have ultimately failed to protect against challenge infection. A better understanding of the correlates of immune protection against HIV infection would greatly assist efforts to develop an effective HIV vaccine [13,14].

**Figure 1:**
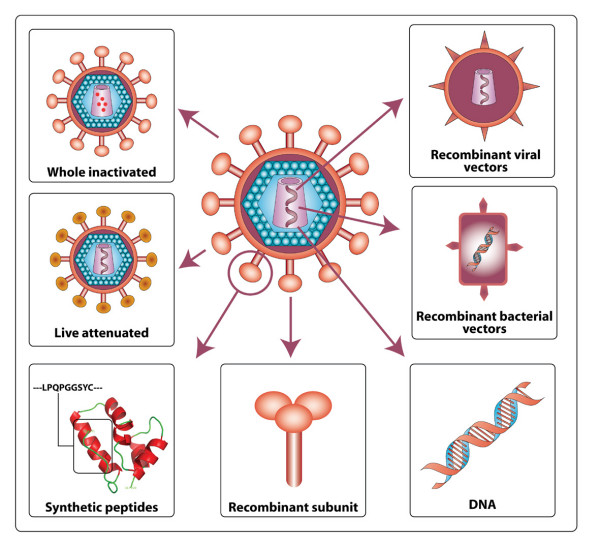
**Various approaches for HIV vaccine development**. The various approaches used in past and present HIV vaccine strategies that are summarized here have been described in detail previously [1-3, 15].

In addition to envelope and DNA prime-boost vaccines, various other strategies have been adopted in HIV vaccine development including the use of recombinant viral and bacterial vectors, synthetic peptides, live attenuated virus, and whole inactivated HIV particles. These strategies have been reviewed in detail recently [1-3,15], and are summarized in Figure 1. Other innovative vaccine strategies that have been recently explored include the use of peptide-loaded dendritic cells [16], and non-infectious viral particles surface-engineered to produce antigen presenting particles that mimic antigen presenting cells [17] to induce cellular immune responses. To date, sterilizing immunity to primate lentiviruses has only been achieved by the use of live attenuated simian immunodeficiency virus (SIV) and chimeric simian-HIV (SHIV) vaccines carrying major deletions in the *nef *gene and other accessory genes [18-21]. Passive infusion of broadly-neutralizing monoclonal antibodies in HIV animal models have also been shown to confer complete protection against challenge infection [22-25]. This provides proof of principle that protection against infection is possible with use of the appropriate antigen. However, *nef*-deleted virus is unlikely to be considered safe enough for use as a HIV vaccine, either because immunization may pose an immediate risk to individuals with weak immune systems, or because the attenuated vaccine strain could eventually evolve to a more pathogenic form [14]. Both of these outcomes have been demonstrated in macaque studies, whereby some animals vaccinated with *nef*-deleted SIV progressed to AIDS in the absence of wild type virus challenge infection [26,27]. Moreover, some individuals infected with *nef*-deleted HIV strains eventually experience CD4+ T-cell loss after many years of asymptomatic infection [28-31]. Nonetheless, studies of long-term survivors (LTS) naturally "vaccinated" with *nef*-deleted HIV, such as the Sydney blood bank cohort (SBBC) [32] and other rare cohorts [33-37], may provide unique insights into protective antibody and CTL responses, which may assist HIV vaccine development [14].

### Epidemiology and Clinical History of the Sydney blood bank cohort

The SBBC consists of 8 individuals (subjects C98, C54, C49, C64, C18, C135, C83 and C124) who became infected with an attenuated strain of HIV via contaminated blood products from a common blood donor (subject D36) between 1981 and 1984 [30,32,38]. The SBBC blood transfusion recipients have been referred to as recipients 7, 13, 12, 9, 10, 4, 8, and 5, respectively, in one previous study [30] and subjects A (C18), B (C64), C (C98), D (C54), E (C49) and F (C83) in an earlier publication [38]. Viral attenuation has been attributed to gross deletions in the *nef*/long terminal repeat (LTR) region of the HIV genome [32]. The clinical history and laboratory studies of the subjects from the first identification as SBBC members through 1998 has been described previously [30], and a detailed update of the clinical and laboratory data through 2006 has been described recently [39]. Briefly, despite being infected from a single source, SBBC members now comprise slow progressors (SP) who have eventually experienced decline in CD4 T-cells after many years of asymptomatic infection (subjects D36, C98, C54), and "elite" long-term nonprogressors (LTNP) who have had stable CD4 T-cell counts and low or below detectable plasma HIV RNA levels for more than 20 years without antiretroviral therapy (ART) and remain asymptomatic (subjects C49, C64, C135) [28,30,31]. Five SBBC members have died of causes either unrelated to- or not directly related to HIV infection (C98, C54, C18, C83, C124) (Table 1). The SBBC therefore provides a unique opportunity to study the pathogenesis of, and immune responses to *nef*-deleted HIV infection in a naturally occurring human setting.

**Table 1:** Clinical history of SBBC members

**Subject**	**Sex**	**Date of Birth**	**Date Transfused**	**Antiretroviral Drugs**^a^	**Clinical History and other information**^a^
D36	M	6/4/1958	N/A; infected with HIV-1 sexually, 12/1980	ABC, AZT, NVP (1/1999-9/2004) ABC, NVP, 3TC (9/2004-present)	Diagnosed with moderate HIVD, 12/1998; SP.
C98	M	7/11/1937	1/2/1982	D4T, NVP, IND (11/1999-death)	Prednisone since 1995 for asthma; died 3/30/2001 from bronchial amyloidosis; death not related to HIV; SP.
C54	M	2/17/1928	7/24/1984	None	IDDM; HCV; surgery for colon cancer in 1995; died 8/28/2001 from myocardial infarct; death not related to HIV; SP.
C49	F	6/9/1954	6/11/1984	None	Diagnosed with age-onset diabetes in 2004, managed by diet; chronic alcoholism; LTNP.
C64	F	3/20/1926	5/4/1983	None	Hypertension; hypercholesterolemia; LTNP.
C135	M	2/23/1946	2/11/1981	None	CCR5Δ32 heterozygote; HLA-B57 positive; LTNP.
C18	M	10/12/1912	8/31/1983	None	Severe coronary atherosclerosis; died 11/1995 from bacterial pneumonia; death not related to HIV; LTNP.
C83	F	12/21/1964	12/30/1982	None	Prednisone since 1982 for SLE; intermittent cyclophosphamide, azathioprine, hydrocortisone; died from combined PCP and pneumococcal pneumonia 4/1987; uncertain if death was HIV related; HIV Progression status uncertain
C124	F	9/30/1917	4/29/1981	None	Died from metastatic gastric cancer 10/1994. Death not related to HIV; HIV Progression status uncertain.

Dates shown are day/month/year. M, male; F, female; ABC, abacavir; AZT, zidovudine; NVP, nevirapine; 3TC, lamivudine; N/A, not applicable; HIVD, HIV associated dementia; SP, slow progressor; LNTP, long-term nonprogressor; IDDM, insulin-dependent diabetes melitis; SLE, systemic lupus erythematosus. ^a^These data have been reported previously [30, 39].

### HIV isolates and viral phenotypes

To determine whether changes in viral phenotype were occurring in SBBC members, HIV isolation was attempted from peripheral blood mononuclear cells (PBMC) collected longitudinally from all subjects except C124 and C83 [28,40], by selected PBMC coculture techniques [40,41] (Table 2). For subjects with detectable but low HIV RNA levels (D36, C54, C98, C18), more than 10 HIV isolates were obtained from each of D36, C54 and C98 over a 5 to 6 year period between November 1994 and November 2000 [40]. Three HIV isolates were obtained from C18 over an 8 month period between July 1993 and March 1994. For subjects with consistently undetectable HIV RNA levels (C49, C64, C124, and C135), a single isolate was obtained from C64 from PBMC collected in February 1996. This was despite isolation attempts from 16 additional PBMC collections between November 1995 and March 2001 [40]. All isolates carried similar but distinct deletion mutations in the *nef *gene and LTR region [28,29,32,42], and were unable to synthesize Nef proteins detectable by Western blotting or immunofluorescence staining of infected cells (D. McPhee and A. Greenway, unpublished data). No isolates were obtained from longitudinal samples of PBMC collected from C49 or C135 over a 4 to 7 year period between February 1994 and October 2000, or from a single sample of PBMC obtained from C124 in March 1993 [40]. Thus, success of isolating *nef*-deleted HIV from SBBC members was strongly dependent on the presence of detectable plasma HIV RNA levels, with few exceptions.

**Table 2:** Phenotypes of *nef*-deleted viruses isolated from SBBC members, and corresponding laboratory data

**Subject**	**Virus isolate**	**Date**	**Years post-infection**	**CD4 cells (cells/μl)**	**HIV RNA (copies/ml)**	**Replication in PBMC**	**Coreceptor usage**
D36	D36II	6/5/95	14.4	N/A	1400	++	CCR5, CXCR4, (CCR2b)
	D36III	8/2/96	15.2	609	1100	++	NT
	D36IV	10/4/96	15.3	504	7700	++	NT
	D36V	9/7/96	15.6	414	2600	++	NT
	D36VI	23/10/96	15.8	432	1100	++	NT
	D36VII	30/1/97	16.1	361	3200	++	NT
	D36VIII	20/5/97	16.4	540	4000	++	CCR5, CXCR4, (CCR2b)
	D36IX	23/12/97	17.0	390	7500	++	CCR5, CXCR4, (CCR2b)
	D36X	15/7/98	17.6	325	N/A	++	NT
	D36XI	22/1/99	18.1	N/A	N/A	++	CCR5, CXCR4, (CCR2b)
	D36XVI	8/11/00	19.9	N/A	BD	++	CCR5

C18	C18(2)	26/7/93	9.8	N/A	N/A	+++	CCR5, (CCR3, Gpr15)
	C18(3)	14/10/93	10.1	N/A	N/A	+++	NT
	C18(4)	7/3/94	10.5	N/A	2804	+++	CCR5, (CCR3, Gpr15)

C54	C54III	7/11/94	10.3	2006	8200	+/-	CCR5, (CCR2b, CCR3)
	C54IV	21/6/95	10.9	1504	3000	+/-	NT
	C54 V	20/12/95	11.4	1054	400	+/-	NT
	C54VI	4/3/96	11.7	1188	1500	+/-	NT
	C54VII	19/6/96	11.9	972	3600	+/-	NT
	C54VIII	16/9/96	12.2	1120	1800	+/-	CCR5, (CCR2b, CCR3)
	C54X	3/3/97	12.7	882	3400	+/-	NT
	C54XI	14/5/97	12.8	1286	N/A	+/-	NT
	C54XII	11/8/97	13.1	1419	1700	+/-	NT
	C54XIII	17/11/97	13.3	1054	N/A	+/-	NT
	C54XIV	5/5/99	14.8	1288	1200	+/-	CCR5, (CCR2b, CCR3)
	C54XV	6/3/00	15.7	840	1600	+/-	NT

C98	C98II	7/12/94	12.9	426	1000	++	CCR5, (CCR3)
	C98III	9/10/95	13.8	576	670	++	NT
	C98IV	7/2/96	14.1	435	200	++	NT
	C98V	22/5/96	14.3	693	290	++	NT
	C98VI	7/8/96	14.6	512	330	++	CCR5, (CCR2b, CCR3)
	C98VII	4/11/96	14.8	646	690	++	NT
	C98VIII	31/1/97	15.0	629	770	++	NT
	C98IX	7/5/97	15.3	529	760	++	NT
	C98X	27/8/97	15.6	612	170	++	NT
	C98XI	26/11/97	15.8	400	N/A	++	NT
	C98XII	30/9/98	16.7	N/D	N/A	++	NT
	C98XIII	3/3/99	17.2	476	N/A	++	NT
	C98XIV	9/11/99	17.8	585	BD	++	CCR5, (CCR2b, CCR3)

C64	C64IV	28/2/96	12.8	850	BD	+/-	CCR5

Dates shown are day/month/year. CD4 cells were measured by flow cytometry. Plasma HIV-1 RNA was measured by COBAS Amplicor HIV-1 Monitor Version 1.0 (Roche Molecular Diagnostic Systems, Branchburg, N.J.) prior to July 1999 and Version 1.5 after July 1999. HIV-1 RNA levels < 400 copies/ml (Version 1) or < 50 copies/ml (Version 1.5) were considered below detection. BD, below detection; N/A, not available; NT, not tested. +++, replication kinetics similar to wild type primary HIV strains; ++, reduced levels of replication and/or delayed replication kinetics compared to wild type primary HIV strains; +/-, levels of HIV replication barely detectable or not detectable by RT assays, but detectable by measurement of extracellular p24 antigen [40].

When compared with wild type HIV isolates and isogenic controls with mutations in *nef*, replication capacity of SBBC isolates in PHA-activated PBMC was found to be consistent over time by viruses isolated from a particular subject, but heterogenous between subjects and fell into 3 distinct phenotypes [28,40] (Table 2). Viruses isolated from C18 replicated rapidly to high levels similar to wild type HIV; viruses isolated from D36 and C98 replicated to lower levels; and viruses isolated from C54 and C64 were barely able to replicate to detectable levels. In contrast, all isolates replicated to equivalent levels in the Cf2-luc reporter cell line [41,43,44] expressing CD4, CCR5 and CXCR4. Thus, SBBC isolates except those from C18 appear to have attenuated replication capacity in PHA-activated PBMC. Inhibiting the *in vivo *replication of HIV in D36 by HAART demonstrated a prolonged *in vivo *virion half life, with a first-phase slope of decline of HIV RNA 0.18/day [45] which is slower than that seen in all previously studied individuals infected with wild-type HIV after commencement of ART [46-49]. Thus, the replication kinetics of D36 virus appears to be attenuated both *in vitro *and *in vivo*.

Analysis of coreceptor usage in transfected Cf2-Luc cells [41] showed that all isolates used CCR5 (R5) as the primary coreceptor for HIV entry, except viruses isolated from D36 prior to commencement of HAART which were dual tropic and used CCR5 and CXCR4 (R5X4) [28,40] (Table 2). These results showed that *nef*-deleted HIV was capable of undergoing a coreceptor switch from R5 to R5X4 *in vivo*. An isolate obtained from D36 after commencement of HAART was CCR5-restricted and had features of an early archived HIV variant, but was genetically similar to HIV present in a CSF sample obtained from D36 during HIV-associated dementia (HIVD) [28]. Thus, for D36, HIV present in CSF during HIVD was likely to be an early variant that underwent compartmentalized evolution in the CNS. Moreover, we showed for the first time that *nef*-deleted HIV is inherently capable of undergoing compartmentalized evolution in the CNS and causing neurologic disease in humans [28]. Stepwise quasispecies diversity was observed in SBBC SP, whereas C49 displayed stable quasispecies diversity most similar to early variants in the SBBC (B. Herring et al., manuscript submitted). Extended analysis of alternative coreceptor usage showed that D36 and C54 isolates could use CCR2b, C18 and C54 isolates could use CCR3, and C18 isolates could use Gpr15 for HIV entry, albeit at low levels [40] (Table 2). Whether expanded usage of alternative HIV coreceptors by SBBC isolates contributes to HIV pathogenesis in these individuals is uncertain, but the unique signature of coreceptor usage for viruses isolated from different SBBC members suggests independent evolution for each virus after infection of each cohort member. This interpretation is consistent with results of Env heteroduplex tracking assays, Env heteroduplex mobility assays and Env V1V2 length polymorphism assays which also demonstrated independent evolution of HIV Env in each subject ([50], and B. Herring et al., manuscript submitted).

### Changes in HIV pathogenicity

To better understand changes in pathogenicity which may have contributed to HIV progression in D36, Jekle et al [51] used an *ex vivo *human lymphoid cell culture system to analyze the ability of 2 HIV viruses isolated from D36 to deplete CD4+ T-cells; one isolated in 1995 prior to the onset of AIDS (D36II) and another isolated in 1999 after the onset of disease progression (D36XI) (Table 2). Although both D36 isolates were less potent in depleting CD4+ T-cells than reference X4 and R5X4 isolates with intact *nef *genes, the 1999 isolate induced greater levels of CD4+ T-cell cytotoxicity than the 1995 isolate. Differences in CD4+ T-cell cytotoxicity between the 2 isolates were evident in CD4+/CCR5- cells, but not evident in CD4+/CCR5+ cells suggesting an increased ability to use CXCR4 by the 1999 isolate. Further studies with the CXCR4 inhibitor AMD3100 showed that, although both isolates were functionally R5X4 [28,40] (Table 2), the 1999 isolate had preferential use of CXCR4 whereas the 1995 isolate had preferential use of CCR5 for HIV entry. These studies showed evolution of R5X4 strains in D36 to a variant with higher cytopathic potential that was associated with increased use of CXCR4 *in vitro *and HIV progression *in vivo*.

Consistent with results of the study by Jekle et al [51], we showed alterations in HIV cytopathicity by sequential D36 isolates in cultures of monocyte-derived macrophages (MDM). Compared with the highly macrophage tropic R5 ADA and R5X4 89.6 isolates, all D36 viruses replicated in MDM to low levels and had delayed replication kinetics [52]. There was no evidence of increased HIV replication in MDM by virus isolated from D36 after HIV progression. However, in support of the results obtained by Jekle et al [51], the 1999 isolate caused extensive cytopathicity in MDM similar to that present in cultures infected with ADA or 89.6, characterized by the presence of many syncytia [52]. In contrast, earlier D36 isolates caused only few or occasional syncytia in MDM despite all D36 viruses replicating in MDM to similar levels. Thus, increased cytopathicity in MDM by the 1999 D36 isolate is most likely due to intrinsic pathogenic features of the Env that increase fusogenicity, similar to that which has been observed by particular neurotropic R5 and R5X4 viruses [53-55]. The increased Env fusogenicity may have contributed to greater cytopathicity by the 1999 D36 isolate and HIV progression in D36. Further studies to elucidate the molecular determinants of D36 Env that are associated with increased fusogenicity are in progress.

### T-cell pathogenesis

The effect of long-term infection with *nef*-deleted virus on CD4+ T cells was studied in detail for six SBBC members [56]. Careful comparison with age- and transfusion-matched controls revealed the surprising result that SBBC members had an increased number of circulating CD45RO+ memory CD4+ T cells. This was unexpected, since these CD4+ T cells are widely believed to represent the main target of cytopathic HIV infection [57-60] (reviewed in [61]), and loss of these cells ultimately leads to acquired immunodeficiency. Therefore, this result is consistent with the hypothesis that *nef*-deleted HIV has reduced pathogenicity *in vivo*.

Nevertheless, in the SBBC subjects studied with detectable plasma viral load, C54 and C98, there was concomitant elevation of CD8+ T cell activation, whereas the SBBC subjects with undetectable plasma viral load, C49, C64 and C135 had normal levels of CD8+ T cell activation [56]. Therefore, within the SBBC, the situation was similar to the strong correlation seen between plasma viral load and CD8+ T-cell activation in subjects infected with wild type HIV [62]. Furthermore, as described above, subjects D36, C98 and C54 exhibited a clear CD4+ T cell decline, albeit at a relatively slow rate [28-30]. This interesting finding argues that pathogenicity within the SBBC was more closely correlated with levels of viral replication (as assessed by plasma viral load) and CD8+ T-cell activation than with viral pathogenicity dictated by the presence or absence of *nef*. This finding likely represents the ability of host factors to modulate the pathogenicity of *nef*-deleted HIV-1 [63,64]. CD8+ T cell activation may reflect lymphocyte turnover during HIV infection, which has been proposed to lead to disruption of normal homeostasis and eventual consumption of both memory and naïve CD4+ T cells [65,66]. However, we did not find evidence of dramatically increased CD4+ T cell turnover in these subjects, as determined by expression of Ki-67 as a marker of cell proliferation [56].

### Evolution of *nef*/LTR sequence

**Figure 2:**
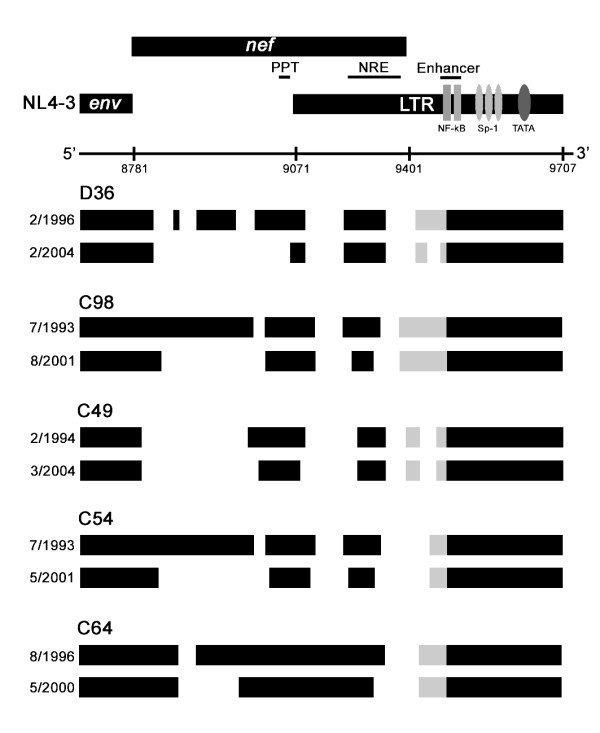
**Convergent evolution of SBBC *nef*/LTR sequences**. Comparisons of the genomic structures of the *nef*/LTR sequences cloned from the earliest available and most recently obtained PBMC samples of D36, C98, C49, C54 and C64 are shown. The genomic structures are compared to wild type HIV (NL4-3). Numbers refer to nucleotide positions in NL4-3. Black boxes represent intact sequence, and gaps represent deletions. Grey blocks represent sequence areas containing alterations of NF-κB and Sp-1 binding sites in the LTR. The dates shown represent the times when PBMC were collected for analysis. PPT, polypurine tract; NRE, negative regulatory unit. This figure has been published previously [29], and is reproduced here with permission from the American society for microbiology.

To determine whether an evolving *nef*/LTR sequence contributed to HIV progression in D36 and C98, we undertook a detailed longitudinal analysis of *nef *and LTR sequence changes occurring in D36, C98, C49, C54 and C64 over a 4 to 10 year period [29]. Sequential analysis of *nef*/LTR demonstrated a gradual loss of *nef *sequence that differed in magnitude between subjects. A large deletion of 128 bp emerged in D36 effectively removing the entire *nef *gene with the exception of the region surrounding the *nef *start codon, the polypurine tract which contains terminal signals for HIV integration, and a 90 bp region of the *nef*/LTR overlap region surrounding the negative regulatory element. The pattern of *nef*/LTR sequence loss in C98 was remarkably similar to that of D36. The pattern of *nef */LTR sequence loss in C54 was also similar, but less extensive than that observed in D36 and C98. However, the additional loss of *nef*/LTR sequence in C64 was comparatively minimal. These data are illustrated in Figure 2, where the *nef*/LTR sequences cloned from the earliest available and most recent blood samples of these subjects are compared. A more detailed longitudinal analysis of *nef*/LTR sequences in these subjects has been reported in Churchill et al [29]. Thus, viruses harboured by D36, C54, C98 and C64 appeared to be evolving in a convergent fashion toward a highly deleted, minimal *nef*/LTR structure containing only sequence elements that are absolutely essential for HIV replication [29]. The convergent nature of the *nef*/LTR sequence changes implies the presence of strong selection pressures that maintains the ability of defective HIV genomes to persist *in vivo*. The highly evolved *nef*/LTR sequences harboured by D36, C54 and C98 were strikingly similar to those that remained dominant in C49 for at least 10 years (Fig. 2) [29]. Thus, the highly evolved *nef*/LTR structure appears to be stable, and in the case of C49 does not increase pathogenicity. However, taken together the results suggest the *in vivo *pathogenicity of *nef*-deleted HIV harboured by SBBC members is dictated by factors other than those that impose a unidirectional selection pressure on the *nef*/LTR region of the HIV genome. Due to the changes in the *nef*/LTR region, these other presumably host factors become more important in terms of disease outcome. This is exemplified by the marked variation from no disease progression with no detectable virus replication (C49 and C64), to no progression with a low viral load (C54), through to slow progression (D36 and C98).

Reversion to pathogenicity by *nef*-deleted SIV has been associated with restoration of a truncated Nef protein [26], acquisition of further deletions in the *nef*/LTR overlap region [67], and/or duplications of NF-κB binding sites in the LTR [67]. In contrast to the SIV studies, the *in vivo *evolution of *nef*-deleted HIV in SBBC members was unidirectional toward a smaller *nef*/LTR sequence and the majority of the additional sequence loss was within the *nef*-alone region [29]. Furthermore, none of the clones were capable of encoding Nef. Together, these results suggest improved viral replication by further deleting remnants of the *nef *gene. In addition, the presence of duplicated NF-κB binding sites in the LTR was not associated with clinical status of the SBBC subjects. Therefore, it is likely that viral factors that modulate the *in vivo *pathogenicity of *nef*-deleted HIV will be distinct from those in *nef*-deleted SIV. Interestingly, the unidirectional evolution toward the minimal *nef*/LTR sequence observed in SBBC members was strikingly similar to the pattern of evolution in a slow progressor infected with a *nef*/LTR-deleted variant of HIV circulating recombinant form 01_AE [34]. The convergent pattern of *nef*/LTR evolution among viruses harboured by SBBC members is therefore unlikely to be due to a unique property of the infecting strain, but rather a positive selection that is common across clades.

### Changes in transcriptional activity

Viruses harbored by SBBC members contain unique alterations of NF-κB and Sp-1 binding sites in the LTR that may affect transcriptional activity and thus, replication capacity [28,29,32]. Therefore, we examined the nucleotide sequence and transcriptional activity of *nef*/LTR clones obtained sequentially from D36 blood samples and from D36 CSF obtained during HIVD, to determine whether changes in LTR activity may contribute to neuropathogenesis of *nef*-deleted HIV-1 infection [68]. We found that the transcriptional activity of CSF-derived *nef*/LTR clones was up to 4.5-fold higher than blood-derived clones isolated before and during HIVD when tested under basal, PMA- and Tat-activated conditions. The presence of duplicated NF-κB and Sp-1 binding sites or a truncated *nef *sequence in blood-derived *nef*/LTRs was not sufficient to mediate large increases in transcriptional activity. However, CSF-derived *nef*/LTRs had duplicated NF-κB and Sp-1 binding sites coupled with a truncated *nef *sequence, which formed a regulatory unit that significantly enhanced LTR transcription [68].

Previous studies showed that LTR variants with augmented transcriptional activity enhance replication of HIV [69]. Therefore, to determine whether D36 *nef*/LTRs affect replication capacity of HIV *in vitro*, we produced and characterized full-length chimeric molecular clones of HIV NL4-3 carrying the *nef*/LTR nucleotide sequence of blood-derived D36 *nef*/LTRs or the CSF-derived D36 *nef*/LTR [68]. We examined the capacity of chimeric NL4-3 viruses carrying D36 *nef*/LTRs to replicate in PBMC compared with wild type NL4-3 and the NL4-3ΔNef deletion mutant [70]. Compared to wild type NL4-3, chimeric HIV containing the *nef*/LTR sequence of blood derived D36 viruses had attenuated replication kinetics, similar to NL4-3ΔNef. In contrast, chimeric HIV containing the *nef*/LTR of D36 CSF had enhanced replication capacity compared to wild type NL4-3. Thus, the *nef*/LTR derived from CSF of D36, which had augmented basal, PMA- and Tat-activated transcriptional activity compared to wild type and blood-derived D36 *nef*/LTRs, augmented replication of HIV in PBMC. Together, our results suggest unique features of the CSF-derived *nef*/LTR restore efficient replication capacity of *nef*-deleted HIV in PBMC by enhancing transcription. The results further suggest that *nef *and LTR mutations that augment transcription may contribute to neuropathogenesis of *nef*-deleted HIV.

### Attenuation in other HIV genes

In addition to *nef *and LTR, mutations or polymorphisms in other HIV genes including *gag*, *rev*, *tat*, *vif*, *vpr*, *vpu *and *env *have been detected in SP or LTNP [71-78]. A previous study of HIV *rev *alleles isolated from a subject with long-term nonprogressive HIV infection showed a persistent Leu to Ile change at position 78 in the Rev activation domain which attenuated Rev function and HIV replication capacity [73], providing evidence that defective *rev *alleles may contribute to long-term survival of HIV infection in some patients. A subsequent study of naturally occurring *rev *alleles with rare sequence variations in the activation domain showed variable reductions in Rev activity [79], although it was unclear from this study whether the observed reductions in Rev activity would be sufficient to attenuate HIV replication capacity. Of note, CTL escape mutations in the second coding exon of Tat have been shown to attenuate virus *in vivo*, suggesting that *in vivo *sequence changes in other regulatory HIV-1 genes may potentially affect HIV-1 pathogenesis [80]. Since differences in HIV pathogenicity in SBBC members could not be fully explained by alterations in the *nef*/LTR region [29] or Env phenotype [40,50], we characterized dominant HIV-1 *rev *alleles that persisted in SBBC LTNP (C18, C64) and SP (C98, D36) [81]. We found that the ability of Rev derived from D36 and C64 to bind the Rev responsive element (RRE) in RNA binding assays was reduced by approximately 90% compared to Rev derived from NL4-3, C18 or C98. D36 Rev also had a 50–60% reduction in ability to express Rev-dependent reporter constructs in mammalian cells. In contrast, C64 Rev had only marginally decreased Rev function despite attenuated RRE binding. In D36 and C64, we found that attenuated RRE binding was associated with rare amino acid changes at 3 highly conserved residues; Gln to Pro at position 74 immediately N-terminal to the Rev activation domain, and Val to Leu and Ser to Pro at positions 104 and 106 at the Rev C-terminus, respectively. In D36, reduced Rev function and altered replication capacity was mapped to an unusual 13 amino acid extension at the Rev C-terminus. However, database analysis of *rev *sequence demonstrated that the presence of one or more of these rare amino acid changes was not able to discriminate between subjects with progressive or non-progressive HIV-1 infection. Moreover, none of these amino acid changes occurred in a previously identified LTNP with defective *rev *alleles [73]. Thus, our studies suggest the contribution of any or all of these mutations to decreased RRE binding and/or attenuated Rev function by SBBC Revs, and possibly to slow or absent HIV progression, is likely to be context dependent.

It is presently unclear whether attenuated D36 Rev function *in vitro *equates to attenuated Rev function *in vivo*, and indeed whether attenuated Rev function contributed to slow progression of HIV infection in this subject. Extrapolation of these *in vitro *findings to an *in vivo *role for attenuated D36 *rev *alleles is difficult, since this subject and other SBBC members are infected with virus containing gross *nef*/LTR deletions which have been shown to cause significant viral attenuation in this cohort [28,29,32]. Nonetheless, our findings raise the possibility that attenuated Rev function may contribute, at least in part, to viral attenuation and slow HIV progression in D36.

### Anti-HIV Ig responses

To better understand the humoral immune response to *nef*-deleted HIV infection, we measured the total IgG responses in longitudinal plasma samples of SBBC members by Western blotting, and compared these with total IgG responses in a control group of LTNP with intact *nef *genes [40]. We found a good correlation between total IgG responses in SBBC members and a detectable plasma VL, with plasma from C18, C54, D36 and C98 all being strongly reactive. Subjects C49 and C64, who consistently maintained undetectable HIV RNA copy numbers [29,30], had significantly reduced total IgG responses. Furthermore, subject C135, who has also had consistently undetectable HIV RNA levels has not fully seroconverted after more than 20 years of infection [40,42]. These studies highlight the importance of adequate antigenic stimulation by *nef*-deleted HIV to drive antibody production. In contrast, we found that total IgG responses in the control group were uniformly potent, reflecting the fact that all these individuals had detectable VLs. Among SBBC members, the strongest antibody responses were observed in individuals with low but detectable VL set points, less than approximately 10,000 RNA copies/ml. This is consistent with a recent observation of an undetectable VL and weak, delayed antibody responses in an unrelated individual with *nef*-deleted HIV [34].

Recent studies have highlighted the change in HIV antibody responses with respect to antibody isotype switching after initial infection, in particular IgG_3 _reactivity to p24 [82,83]. Preliminary WB testing of p24 IgG_3 _responses in the SBBC LTNP indicated there was reactivity for those individuals with the most potent total IgG responses (C18 and C54) (E. Verity, D. McPhee, K. Wilson, and D. Zotos, unpublished data). This hints at delayed isotype switching and hence delayed maturation of immune responses for at least some members of the SBBC.

### Neutralization studies of *nef*-deleted HIV

In additional studies, we determined whether plasma from SBBC members had differences in ability to neutralize *nef*-deleted HIV strains. We did this by comparing the ability of longitudinally collected plasma from SBBC members or from control LTNP cohort members to neutralize the infectivity of HIV isolated from D36 and C18 [40]. We found that, for SBBC plasmas, neutralization of D36 or C18 viruses strongly correlated with VL, replication capacity of the isolated virus, and the strength of anti-HIV IgG responses. Plasma from SBBC members with undetectable VL was unable to neutralize the infectivity of these viruses. In contrast, plasmas from the control LTNP cohort were able to neutralize the infectivity of SBBC viruses with titres generally higher than that seen for SBBC members, but there was no correlation between neutralization and HIV RNA copy number or IgG responses in the control LTNP group.

### Broad neutralizing antibody responses

A number of studies have suggested an increased frequency of LTNP and long term survivors (LTS) possess strong, cross-reactive neutralising antibody responses [84-86]. However, very few studies have investigated antibody responses in LTNP/LTS infected with *nef*-deleted HIV, despite a number of studies showing strong neutralising antibody responses in macaques infected with *nef*-deleted SIV [87-92]. However, studies by Greenough et al., [93] showed measurable neutralising antibody responses concurrent with a detectable VL for a single individual infected with *nef*-defective HIV. We therefore determined the breadth of neutralizing antibodies against HIV for SBBC members.

We found that plasma from C18, and to a lesser extent D36, C54 and C98 were able to potently neutralize the infectivity of a number of laboratory adapted and primary HIV strains, but little or no neutralizing activity was evident in plasma from C49, C64 and C135 [40]. Further analysis of cross reacting neutralizing antibodies in SBBC plasma demonstrated that plasma derived from C18 was able to potently neutralize the infectivity of HIV-1_ROK39 _(clade A), HIV-1_SE364 _(clade C), HIV-1_BCB93 _(clade D), and HIV-1_92TH024 _(CRF01_AE), while plasma derived from C98 was able to only weakly neutralize the infectivity of HIV-1_ROK39 _and HIV-1_SE364 _[40].

Together, our studies on antibody responses in SBBC members demonstrated that infection with *nef*-deleted HIV can, in some individuals, induce antibody responses capable of potently neutralizing a broad range of isolates. It is possible that the breadth of antibody responses observed in SBBC members may be associated with unrestricted immunoglobulin class switching, which is inhibited by Nef [94]. This would be counterbalanced by the very low viral replication *in vivo *(VL) and a detectable IgG_3 _p24 response for C18 and C54. Hence, the broad antibody response may also reflect an early response to infection. The strongest antibody responses in SBBC members were observed in individuals with a detectable VL, supporting the model of a VL threshold which must be reached to provide adequate antigenic stimulation to drive antibody production [14]. However, this higher level of virus replication places these individuals at greatest risk of disease progression. Signs of disease progression were observed for 2 SBBC members (D36, C98) [28-30], demonstrating that the potent neutralizing antibody responses observed did not protect from disease progression. These results question the effectiveness of a broad neutralizing antibody response in individuals with established infection. However, this does not preclude an important role for neutralizing antibodies in preventing initial infection.

### T-cell responses

Analysis of cellular immune mechanisms suppressing *nef*-deleted virus in SBBC nonprogressors may provide insights relevant for HIV vaccine design. Several studies have demonstrated that the presence of a sustained Gag-specific CD8+ T-cell response is associated with protection against disease progression in cohorts of LTNP infected with *nef*-intact HIV [95-97]. In support, our studies have also shown that the distinguishing feature of LTNP harbouring *nef*-intact HIV is the predominance of Gag-specific CD8+ T-cell responses, which decline when these individuals start to progress (W.B. Dyer et al., manuscript in preparation). However, novel qualitative and quantitative differences in immune correlates of viral control may exist in LTNP infected with *nef*-deleted HIV. Longitudinal studies of T-cell responses in SBBC members have demonstrated a dominance of Pol-specific CD8+ T-cell responses rather than those against Gag [98] and W.B. Dyer et al., manuscript in preparation). This suggests that infection with *nef*-deleted HIV may give rise to a qualitatively different response, although a subset of SBBC members also had strong CD8+ T-cell responses to Gag [98]. Nonetheless, CD8+ T-cell responses to Gag or Pol did not discriminate SBBC LTNP from SP.

**Figure 3:**
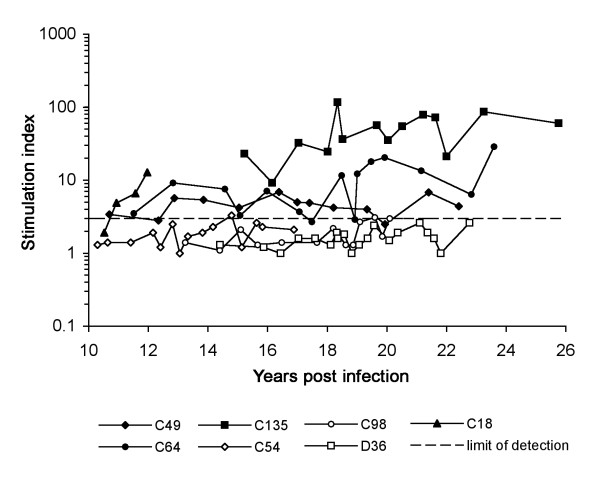
**Longitudinal analysis of T-helper proliferative responses to HIV p24 antigen in SBBC progressors and nonprogressors**. T-helper proliferative responses to HIV p24 antigen were determined by [^3^H]-thymidine incorporation, as described previously [99, 100]. T-helper responses were persistently detectable (stimulation index > 3) in all nonprogressing subjects with below detectable plasma HIV RNA levels (C135, C64, and C49), but absent in all progressors (D36, C98, and C54). Of note, T-helper responses were detectable over a short window in C18, who was a nonprogressing individual with detectable plasma HIV RNA levels.

In contrast, longitudinal analysis of HIV-specific CD4+ T-cells showed that all SBBC nonprogressors able to completely control plasma HIV RNA levels to below detectable levels (C49, C64 and C135) had persistent and strong T-helper proliferative responses to HIV p24 antigen, whereas these responses were absent in all progressors with persistent viremia (C54, C98 and D36) (Fig. 3). The notable exception was C18 who had detectable but low plasma HIV RNA levels without evidence of CD4+ T-cell loss, and had detectable p24 T-helper responses over a short period of approximately 12 months leading up to his non-HIV related death at 83 years of age. In this subject, the p24 T-helper responses coincided with an exponential increase in CTL responses against Pol antigens, measured by memory CTL precursor frequency assay [98], and IFN-gamma ELISPOT responses (W. Dyer, unpublished data). However, it is difficult to interpret the significance of the T-cell responses in C18 given the short window of analysis. Together, although derived from a small cohort of individuals, these results suggest that immune suppression of *nef*-deleted HIV-1 by SBBC LTNP may be dependent upon persistent T-helper responses irrespective of the CD8+ T-cell and neutralizing antibody response to viral antigens.

## Conclusion

The development of an effective HIV vaccine has been hampered by the lack of defined correlates of immune protection against HIV infection. Although *nef*-deleted strains of HIV are not suitable as live attenuated HIV vaccines due to safety concerns, the only lentiviral vaccines to date that have generated sterilizing immunity in animals are those based on live, attenuated viruses. To this end, studies of SBBC members naturally "vaccinated" with *nef*-deleted HIV may provide unique insights into protective immune responses to HIV infection, which may assist the development of an effective HIV vaccine. As a consortium, we have been studying *nef*-deleted HIV infection in the SBBC for a number of years, linking changes in pathogenicity *in vivo *with viral evolution and immune responses to infection. The major aim of this review was to summarize our recently published and unpublished studies on the SBBC. Together, the studies show that potent, broadly neutralizing anti-HIV antibodies and robust CD8+ T-cell responses to HIV infection were not necessary for long-term control of HIV infection in a subset of SBBC members, and were not sufficient to prevent HIV sequence evolution, augmentation of pathogenicity and eventual progression of HIV infection in another subset. However, a strong T-helper proliferative response to HIV p24 antigen was associated with long-term control of infection. Variation in the outcome of HIV infection in this cohort appears to be strongly host-dependent, consistent with other studies with wild-type HIV. Dependence upon the host's unique immune environment also appears to be important in contributing to control of infection. This further complicates development of a successful vaccine. We hope that results gleaned thus far from studies of this unique cohort of individuals will provide HIV vaccine researchers with novel insights into immune mechanisms that may serve to prevent or control HIV infection.

## Competing interests

The author(s) declare that they have no competing interests.

## Authors' contributions

The SBBC project is a multicenter consortium. PRG, DAM, JL, JSS, SMC, JM, BJB, ALC and MJC are principal SBBC investigators who, along with SRL contributed to the study design, analysis and interpretation of the data. EV performed the neutralization studies and helped determine the viral phenotypes. WBD and JJZ performed the T-cell experiments. MJC performed the *nef *cloning and sequencing. MR provided technical expertise and contributed intellectually. DG performed the viral phenotyping in conjunction with PRG. PRG wrote the manuscript. All authors helped edit the manuscript. All authors have seen and approved the final manuscript.
